# Supplementary material for: Ultrafast enzymatic digestion of deoxyribonucleic acid in aqueous microdroplets for sequence discrimination and identification
Source: QRB Discov. 2021 May 20;2:e4. doi: 10.1017/qrd.2021.2 (PMC8185430; doi:10.1017/qrd.2021.2)
Supplement: Supplementary file 1 [file S2633289221000028sup001.docx]

Supplementary Information

**Ultrafast Enzymatic Digestion of Deoxyribonucleic Acid in Aqueous**

**Microdroplets for Sequence Discrimination and Identification**

Xiaoqin Zhong1,2, Hao Chen3, Richard N. Zare1,4*

1Department of Chemistry, Fudan University, Shanghai 200438, China

2Department of Environment and Chemical Engineering, Shanghai University, Shanghai 200444, China

3Department of Chemistry & Environmental Science, New Jersey Institute of

Technology, Newark, NJ, 07102, USA

4Department of Chemistry, Stanford University, Stanford, California 94305, USA

*Corresponding authors:

Richard N. Zare, E-mail: [rnz@stanford.edu](mailto:rnz@stanford.edu)

**Supplementary Table S1:** Digestion extent of different DNA sequences by DNase I

with ESSI-MS. A positive voltage of 3 kV was applied for the digestion and detection.


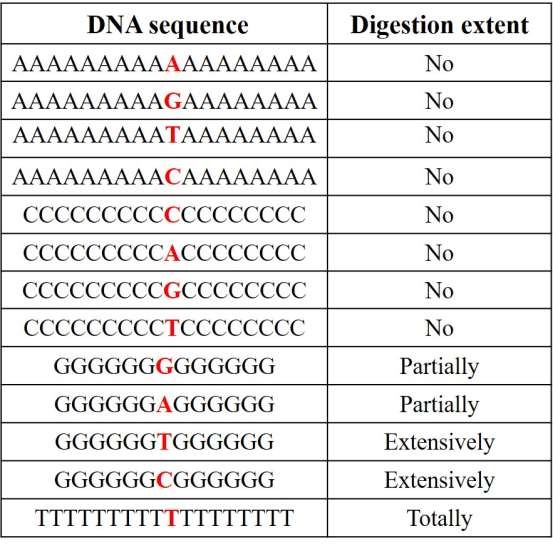


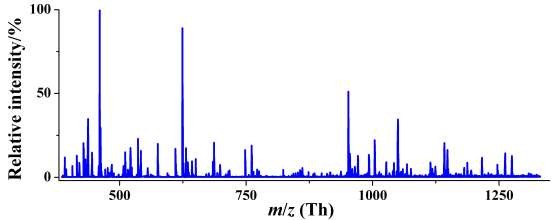


**Supplementary Figure S1:** Mass spectrum of fragments identified from digests of

DNA sequence containing pure T by ESSI-MS applied with a positive voltage (+3 kV).


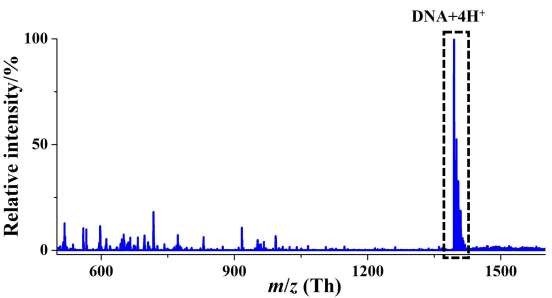


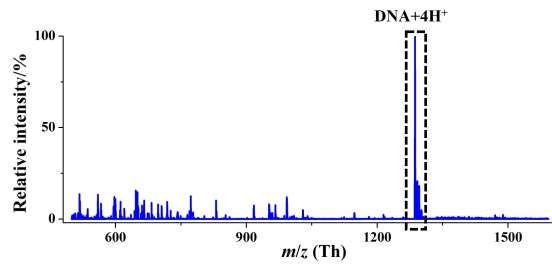
**Supplementary Figure S2:** Mass spectrum of fragments identified from digests of DNA sequence containing pure A incubated in bulk phase overnight at room temperature. ESSI-MS was applied with a positive voltage (+3 kV) for detection.

**Supplementary Figure S3:** Mass spectrum of fragments identified from digests of DNA sequence containing pure C incubated in bulk phase overnight at room temperature. ESSI-MS was applied with a positive volta
